# Ginger Extract Ameliorates Obesity and Inflammation via Regulating MicroRNA-21/132 Expression and AMPK Activation in White Adipose Tissue

**DOI:** 10.3390/nu10111567

**Published:** 2018-10-23

**Authors:** Seunghae Kim, Mak-Soon Lee, Sunyoon Jung, Hye-Yeon Son, Seonyoung Park, Bori Kang, Seog-Young Kim, In-Hwan Kim, Chong-Tai Kim, Yangha Kim

**Affiliations:** 1 Department of Nutritional Science and Food Management, Ewha Womans University, 52 Ewhayeodae-gil, Seodaemun-gu, Seoul 03760, Korea; shkyr1120@naver.com (S.K.); troph@hanmail.net (M.-S.L.); cococosy@naver.com (S.J.); shyfree@gmail.com (H.-Y.S.); rain9125@naver.com (S.P.); pasodi00@naver.com (B.K.); saraha9390@gmail.com (S.-Y.K.); 2 Department of Integrated Biomedical and Life Sciences, Korea University, Seoul 02841, Korea; k610in@korea.ac.kr; 3 Research Group of Bioprocess Engineering, Korea Food Research Institute, Wanju-gun, Jeollabuk-do 55365, Korea; ctkim@kfri.re.kr

**Keywords:** ginger extract, obesity, inflammation, microRNA-21, microRNA-132, AMPK

## Abstract

Ginger is a plant whose rhizome is used as a spice or folk medicine. We aimed to investigate the effect of ginger root extract on obesity and inflammation in rats fed a high-fat diet. Sprague-Dawley rats were divided into three groups and fed either a 45% high-fat diet (HF), HF + hot-water extract of ginger (WEG; 8 g/kg diet), or HF + high-hydrostatic pressure extract of ginger (HPG; 8 g/kg diet) for 10 weeks. The HPG group had lower body weight and white adipose tissue (WAT) mass compared to the HF group. Serum and hepatic lipid levels of HPG group were lower, while fecal lipid excretion of the HPG group was higher than that of the HF group. In the WAT of the WEG and HPG groups, mRNA levels of adipogenic genes were lower than those of the HF group. Moreover, HPG group had lower mRNA levels of pro-inflammatory cytokines than did the HF group. MicroRNA (miR)-21 expression was down-regulated by both WEG and HPG. Additionally, miR-132 expression was down-regulated by HPG. The adenosine monophosphate-activated protein kinase (AMPK) activity of HPG group was greater than that of the HF group. HPG may have beneficial effects on obesity and inflammation, partially mediated by regulation of miR-21/132 expression and AMPK activation in WAT.

## 1. Introduction

Obesity refers to excessive body fat accumulation. It induces systemic low-grade inflammation, which increases the risk of type 2 diabetes, hypertension, cardiovascular diseases, and certain cancers. Obesity-induced inflammation occurs because of the continuous lipid accumulation in adipose tissue [1]. Pro-inflammatory molecules produced by adipose tissue are active participants in the development of insulin resistance and increase the risk of metabolic disease associated with obesity [1]. To address these issues, many studies have been conducted to inhibit lipid accumulation and pro-inflammatory cytokine synthesis using food materials. Typical functional food components such as curcumin, quercetin, resveratrol, *Camellia sinensis*, and green tea were found to alleviate lipid accumulation and inflammation induced by metabolic diseases such as obesity and hypertension [2,3,4].

Ginger (*Zingiber officinale* Roscoe) is a herbaceous plant widely cultivated as a spice or for natural food therapy. In traditional medicine, ginger has been used for diseases such as indigestion, vomiting, joints and muscle pain, and cold [5]. Moreover, its various pharmacological effects have been reported, including anti-obesity, anti-inflammatory, and anticancer effects [6]. The currently known ginger extraction methods are hot water extract, ultra-sonication assisted extract and more [7]. While the heating method has a high possibility of losing functional compounds in food, the high-hydrostatic pressure (HHP) method, a non-thermal food processing technology, extracts functional compounds with higher ease without damaging them by destroying their covalent bonds and cell membrane structures [8].

In this study, we examined the effect of high-hydrostatic pressure extract of ginger (HPG) on high-fat (HF) diet-induced obesity and inflammation, and measured the expression levels of genes involved in adipogenesis and pro-inflammatory cytokines in white adipose tissue (WAT). In addition, we evaluated the microRNA (miR)-21 and miR-132 expression, as well as adenosine monophosphate-activated protein kinase (AMPK) activity in WAT.

## 2. Materials and Methods

### 2.1. Preparation of Materials

HPG and hot water extract of ginger (WEG) were kindly supplied by Korea Food Research Institute (Songnam, Gyeonggi, Korea). The WEG was used as a reference. The ginger root used in the preparation of HPG and WEG was purchased from the local market of Muan (Muan-gun, Jeollanam-do, Korea). Fresh ginger root was added in distilled water, followed by pulverization with a waring blender. The ginger root suspension was used for the preparation of HPG and WEG. The preparation of HPG and WEG were in accordance with the method by Jung et al. [9]. Briefly, the ginger root suspension was poured into plastic bags with 25 mL of each enzyme (Thermamyl 120 L, Celluclast 1.5 L andViscozyme L; Novo Nordisk, Bagsvaerd, Denmark) and transferred to a programmable high-pressure treatment apparatus (TFS-10 L; Innoway Co., Bucheon, Korea) set at a pressure of 100 MPa for 24 h at 50 °C. After incubation, the extract was heated at 100 °C for 10 min to inactivate the enzymes. After cooling, the extract was centrifuged at 11,000× *g* for 10 min. The supernatant was filtered using No. 4 filter paper and the filtrate was freeze-dried and used as HPG. The WEG was prepared as follows [9]: Ginger root suspension was placed into a round-bottom flask fitted with a cooling condenser, and extraction was performed at 100 °C for 3 h. The extract was followed by centrifugation at 11,000× *g* for 10 min, and the supernatant was filtered using No. 4 filter paper. The filtrate was freeze-dried and used as WEG.

### 2.2. Determination of 6-Gingerol, 6-Shogaol and Total Saponin

The 6-gingerol and 6-shogaol were analyzed by high-performance liquid chromatography (HPLC) using a JASCO HPLC system (Tokyo, Japan), according to a method by Moon et al. [10]. Standard 6-gingerol and 6-shogaol were purchased from Sigma-Aldrich (St. Louis, MO, USA).

Total saponin content of each extract was determined using the method described by Moon et al. [10]. The ginsenoside Re (Wako Chem. Co., Osaka, Japan) was used as a reference standard.

### 2.3. Animals and Experimental Design

All experimental procedures were approved by the Institutional Animal Care and Use Committee (IACUC) of Ewha Womans University, Korea (IACUC No. 15-002). Twenty-seven Sprague–Dawley (male, 3-week-old) rats were housed individually in stainless steel wire mesh cages under controlled environment with a temperature of 22 ± 2 °C, humidity of 55 ± 5%, and a 12-h light and dark cycle. After a week of adaptation, the animals were randomly divided into three groups (*n* = 9/group) and fed the following experimental diet for 10 weeks: a high-fat diet (HF), high-fat diet containing WEG (8 g/kg diet), and high-fat diet containing HPG (8 g/kg diet). The diet compositions are shown in supplementary Table S1. During the experimental period, body weight and food intake were measured twice a week using a digital scale. Average daily intake was determined by dividing total food intake by the number of days fed. After fasting for 12 h, the rats were anesthetized with a mixture of Zoletil 50 (Virbac Laboratories, Carros, France) and Rompun (Bayer Korea, Seoul, Korea), and euthanized. Blood was collected by cardiac puncture and serum was separated by centrifugation. The collected serum, liver and epididymal adipose tissue were stored at −70 °C until analysis.

### 2.4. Serum Biochemical Measurements

Serum concentrations of triglyceride (TG), total cholesterol (TC), high-density lipoprotein cholesterol (HDL-C), aspartate transaminase (AST), and alanine transaminase (ALT) were measured based on enzymatic colorimetric method using a commercial kit (Asan pharmaceutical, Seoul, Korea) in accordance with the manufacturer’s instructions. Low-density lipoprotein cholesterol (LDL-C) was calculated by the Friedewald formula (LDL-C (mg/dL) = TC − HDL-C − (TG/5)) [11].

### 2.5. Hepatic and Fecal Lipid Analysis

Hepatic and fecal lipids were extracted using the method of Bligh and Dyer [12] with slight modifications. Levels of TG and TC in the liver and feces were analyzed by the enzymatic colorimetric method described for serum lipid analysis.

### 2.6. Histological Analysis

Epididymal adipose tissue was fixed in 10% formalin solution overnight. The fixed tissue was processed using an automatic tissue processor (TP1020, Leica, Mannheim, Germany). The processed tissues were infiltrated with paraffin (Paraplast Plus, Leica), cut into 7-μm-thick sections, and then stained with hematoxylin and eosin (H&E). Image of H&E sections were obtained using a microscope (Olympus, Tokyo, Japan) at 200× magnification. To determine adipocyte size, H&E staining images were analyzed using Image J software. Thirty cells per sample were included in the analysis for each group.

### 2.7. Quantitative Real-Time PCR (qRT-PCR)

Total RNA was extracted from epididymal adipose tissue using TRIzol reagent (GeneAll Biotechnology, Seoul, Korea) according to the manufacturer’s instructions. cDNA was synthesized from total RNA using a Moloney Murine Leukemia Virus (M-MLV) Reverse Transcriptase kit (Bioneer Co., Daejeon, Korea). Real-time qPCR was performed using Rotor Gene 3000 (Corbett Research, Mortlake, N.S.W., Australia) and AccuPower 2X Greenstar qPCR MasterMix (Bioneer Co., Daejon, Korea). Primers used for real-time qPCR analysis are described in Supplementary Table S2. Data analysis was conducted by the 2^−ΔΔCt^ method. β-actin was used as the reference gene for normalization.

For the analysis of miR expression, cDNA was synthesized using a miRNA cDNA Synthesis Kit with Poly (A) Polymerase Tailing (ABM Inc., Richmond, BC, Canada). The synthesized cDNA was amplified using the EvaGreen miRNA qPCR Master Mix (ABM Inc.). Quantification of miRs was carried out using miR-21, miR-132, and U6 specific primers (ABM Inc). Real-time qPCR amplification was performed using the Rotor Gene 3000 (Corbett Research). Levels of miR-21 and miR-132 were normalized to U6 snRNA and determined using the 2^−ΔΔCt^ method.

### 2.8. AMP-Activated Protein Kinase (AMPK) Activity

AMPK activity was evaluated using an AMPK Kinase Assay kit (Cyclex, Nagano, Japan) according to the manufacturer’s instructions. Protein levels were determined using a bicinchoninic acid (BCA) protein assay kit (Thermo Scientific, Waltham, MA, USA). AMPK activity was normalized to protein concentration and expressed as fold change relative to the control group.

### 2.9. Statistical Analysis

Data are expressed as mean ± standard error of the mean (SEM). The SPSS software (version 22; IBM Corporation, Armonk, NY, USA) was used for the statistical analyses. Data were tested for normal distribution by the Kolmogorov–Smirnov normality test. Then the comparisons between groups were made by one-way analysis of variance (ANOVA) and Tukey’s post hoc multiple comparison tests. *p*-values less than 0.05 were considered statistically significant.

## 3. Results

### 3.1. Contents of 6-Gingerol, 6-Shogaol and Total Saponin of Ginger Extracts

**Figure 1:**
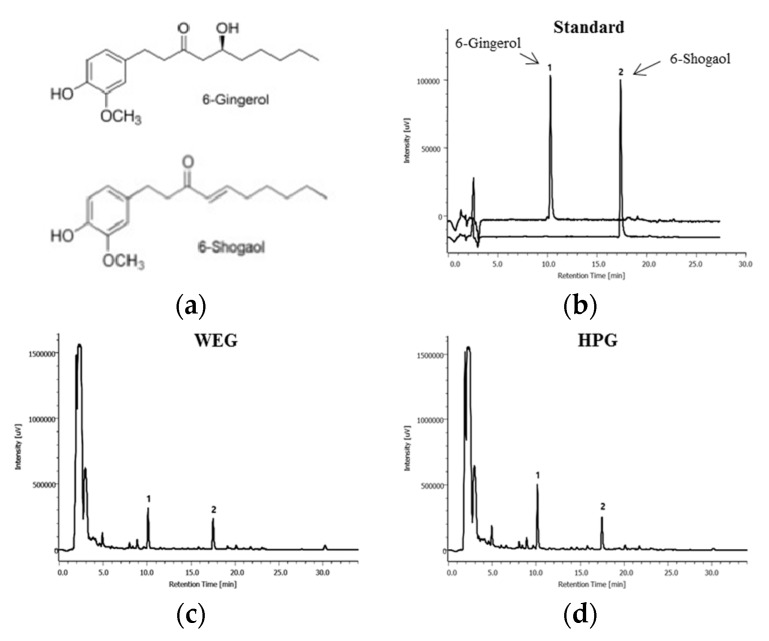
High-performance liquid chromatography (HPLC) analysis of ginger extracts. (**a**) Chemical structures of 6-gingerol and 6-shogaol; HPLC chromatogram of (**b**) standard, (**c**) hot water extract of ginger (WEG), and (**d**) high-hydrostatic pressure extract of ginger (HPG).

The chemical structures of 6-gingerol and 6-shogaol are shown in Figure 1a. The HPLC chromatogram of the 6-gingerol and 6-shogaol is presented in Figure 1b–d.

**Figure 2:**
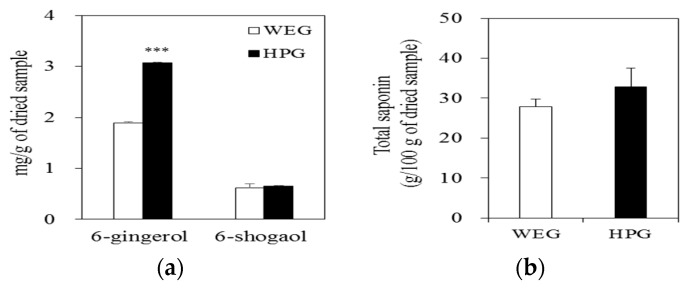
Bioactive compositions of WEG and HPG. (**a**) 6-gingerol and 6-shogaol of WEG and HPG and (**b**) total saponin of WEG and HPG. Values are expressed as mean ± SEM (*n* = 3) of three independent experiments. *** *p* < 0.001 compared to the WEG. WEG: hot water extract of ginger; HPG: high-hydrostatic pressure extract of ginger.

The quantities of 6-gingerol in the WEG and HPG were 1.894 ± 0.02 and 3.067 ± 0.09 mg/g, respectively (*p* < 0.001), and 6-shogaol amounts were 0.620 ± 0.01 and 0.652 ± 0.01 mg/g, respectively (Figure 2a). The total saponin amounts of the WEG and HPG were 27.86 ± 1.97 and 32.89 ± 4.65 g/100 g, respectively (Figure 2b).

### 3.2. Body Weight, Intakes, and Fat Accumulation

**Figure 3:**
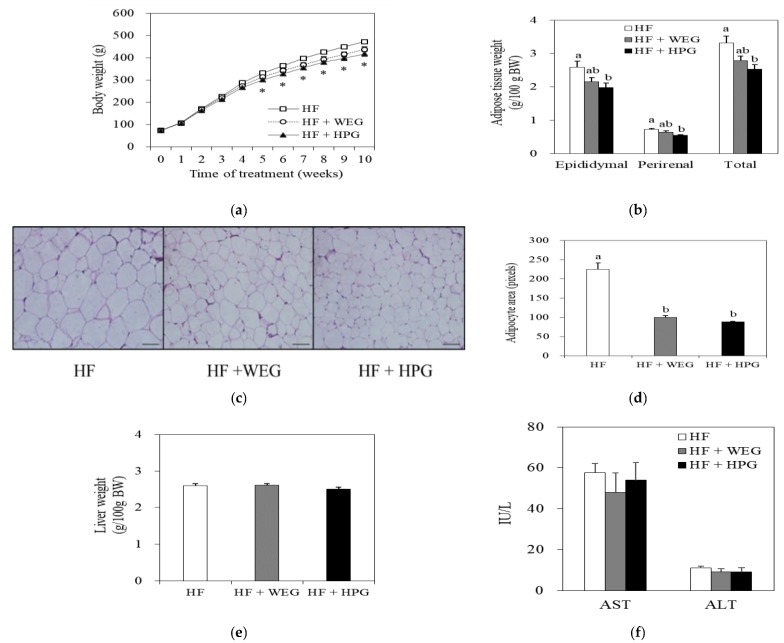
Effects of WEG and HPG on diet-induced obesity. (**a**) Body weight and (**b**) adipose tissue weight of rats fed WEG and HPG diets for 10 weeks; (**c**) Representative histological sections of epididymal adipose tissue; hematoxylin and eosin stain, scale bar = 100 μm; (**d**) Average adipocyte size was presented as pixels; (**e**) Liver weight and (**f**) serum aspartate transaminase (AST) and alanine transaminase (ALT) levels. Values are expressed as mean ± SEM (*n* = 9/group). * *p* < 0.05 compared to the HF group. ^a,b^ Mean values with unlike superscript letters are statistically different at *p* < 0.05. HF: high fat; WEG: hot water extract of ginger; HPG: high-hydrostatic pressure extract of ginger.

The initial body weights were not significantly different among the three groups. At 10 weeks of treatment, the body weight and body weight gain of HPG group were 11.5% and 13.4% lower than those of the HF group (*p* < 0.05) (Table 1 and Figure 3a).

During the experimental period, food efficiency and energy efficiency in the HPG groups were lower than those in the HF group (*p* < 0.05), while food intake and energy intake did not significantly differ among the three groups (Table 1).

The total weight of WAT containing epididymal and perirenal adipose tissue mass of the HPG group was lower than that of the HF group (*p* < 0.05) (Figure 3b). As the representative images of adipose tissue show, the sizes of epididymal adipocytes were significantly smaller in both WEG and HPG groups than those in the HF group (Figure 3c,d).

### 3.3. Liver Weight and Serum AST and ALT Activities

To test whether ginger extracts induce liver toxicity, the liver weight and serum AST and ALT levels were measured. There were no significant differences among the three groups in the relative liver weight and serum AST and ALT levels (Figure 3d,e).

### 3.4. Serum, Liver, and Fecal Lipid Profiles

**Figure 4:**
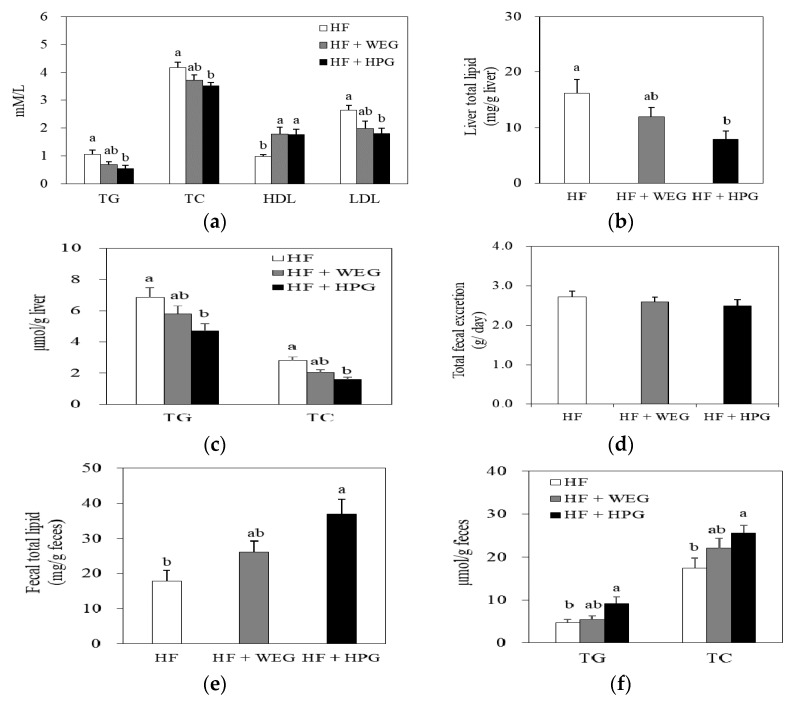
Effects of WEG and HPG on lipid profiles of serum, liver and feces. (**a**) Serum lipid profiles; (**b**, **c**) liver lipid profiles; (**d**) total fecal excretion; and (**e**,**f**) fecal lipid profiles. Values are expressed as mean ± SEM (*n* = 9/group). ^a,b^ Mean values with unlike superscript letters are statistically different at *p* < 0.05. HF: high fat; WEG: hot water extract of ginger; HPG: high-hydrostatic pressure extract of ginger; TG: triglyceride; TC: total cholesterol; HDL-C: high-density lipoprotein cholesterol; LDL-C: low-density lipoprotein cholesterol.

The levels of serum TG, TC, and LDL-C were significantly lower in the HPG group than in the HF group (*p* < 0.05) (Figure 4a). On the other hand, the levels of HDL-C in the WEG and HPG groups was significantly higher than those in the HF group (*p* < 0.05) (Figure 4a).

Hepatic total lipid level of the HPG group was lower than that of the HF group (*p* < 0.05) (Figure 4b). The hepatic TG and TC levels of the HPG group were also lower than those of the HF group (*p* < 0.05) (Figure 4c). In feces, total lipid level in the HPG group was higher than that in the HF group (*p* < 0.05), whereas the total fecal matter excretion amount was not significantly different (Figure 4d,e). Moreover, HPG enhanced the fecal TG and TC excretion compared to the HF group (*p* < 0.05) (Figure 4f).

### 3.5. mRNA Expression of Genes Related to Adipogenesis and Pro-Inflammatory Cytokines in WAT

**Figure 5:**
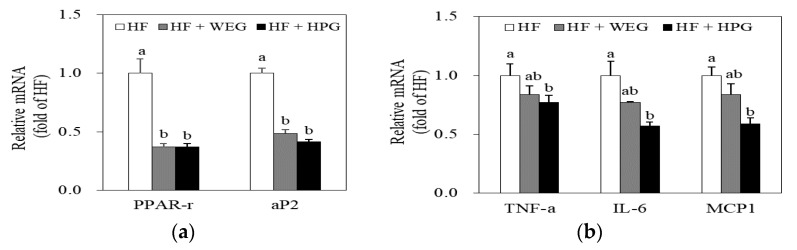
Effects of WEG and HPG on mRNA expression of genes related to adipogenesis and pro-inflammatory cytokines in white adipose tissue (WAT). The mRNA levels of (**a**) adipogenesis and (**b**) pro-inflammatory cytokines were measured using real-time qPCR. Values represent fold changes compared to the control. Values are expressed as mean ± SEM (*n* = 9/group). ^a,b^ Mean values with unlike superscript letters are statistically different at *p* < 0.05. HF: high fat; WEG: hot water extract of ginger; HPG: high-hydrostatic pressure extract of ginger; PPAR-γ: peroxisome proliferator-activated receptor-γ; aP2: adipocyte protein 2; TNF-α: tumor necrosis factor-α; IL-6: interleukin-6; MCP1: monocyte chemoattractant protein-1.

The mRNA expression of genes related to adipogenesis and pro-inflammatory cytokines in WAT were analyzed. The mRNA levels of peroxisome proliferator-activated receptor-γ (PPAR-γ) and adipocyte protein 2 (aP2) were lower in both the WEG and HPG groups than those in the HF group (*p* < 0.05) (Figure 5a). The mRNA levels of tumor necrosis factor-α (TNF-α), interleukin-6 (IL-6), and monocyte chemoattractant protein-1 (MCP-1) were lower in the HPG group than in the HF group (*p* < 0.05), whereas WEG treatment did not affect these levels significantly (Figure 5b).

### 3.6. miR-21 and miR-132 Expression in WAT

**Figure 6:**
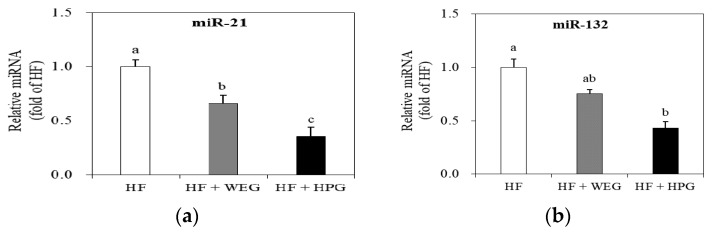
Effects of WEG and HPG on microRNA(miR)-21 and miR-132 in WAT. The levels of (**a**) miR-21 and (**b**) miR-132 were measured using real-time qPCR. Values are expressed as mean ± SEM (*n* = 9/group). ^a,b^ Mean values with unlike superscript letters are statistically different at *p* < 0.05. HF: high fat; WEG: hot water extract of ginger; HPG: high-hydrostatic pressure extract of ginger.

To elucidate the molecular mechanisms underlying the regulation of lipid metabolism and inflammation of HPG, miR-21 and miR-132 expression levels were analyzed in WAT. miR-21 expression in the WEG and HPG groups was down-regulated by 33.9% and 64.1%, respectively, compared to that in the HF group (*p* < 0.05) (Figure 6a). Moreover, the miR-21 level in the HPG group was 45.7% lower than that in the WEG group (*p* < 0.05) (Figure 6a). The expression of miR-132 was 57.1% lower in the HPG group compared to that in the HF group (*p* < 0.05) (Figure 6b).

### 3.7. AMPK Activity in WAT

**Figure 7:**
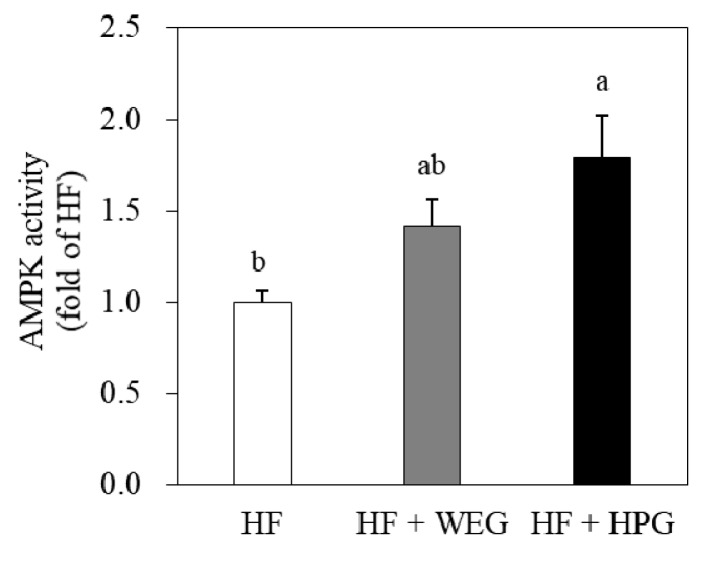
Effects of WEG and HPG on adenosine monophosphate-activated protein kinase (AMPK) activity in WAT. Values are expressed as mean ± SEM (*n* = 9/group). ^a,b^ Mean values with unlike superscript letters are statistically different at *p* < 0.05. HF: high fat; WEG: hot water extract of ginger; HPG: high-hydrostatic pressure extract of ginger.

We determined the activity of AMPK, an important metabolic regulator, which affects the regulation of genes related to adipogenesis and inflammation in WAT. The AMPK activity was significantly enhanced by 1.8-fold in the HPG group compared to the HF group (*p* < 0.05) (Figure 7).

## 4. Discussion

Natural foods have a variety of physiologically active substances that help prevent/treat diseases. High-hydrostatic pressure (HHP) technology has attracted attention as a low-temperature extraction method that does not destroy or denature the active substances by heat during the extraction process of various natural products [8]. Therefore, we evaluated the effects of HHP extract of ginger (HPG) on high-fat diet-induced obesity, as well as the molecular factors involved in lipid metabolism and inflammation of the white adipose tissue (WAT). The dose of the ginger extracts used in the study was well tolerated by rats, demonstrated by the fact that the relative liver weight and serum levels of AST and ALT were unaffected by ginger supplementation. Significant reduction of body weight in the HPG group was observed at 5 weeks after beginning the HPG diet. The total fat mass containing epididymal and perirenal adipose tissue was lower in the HPG group than that in the HF group. These results suggested that HPG efficiently inhibited body weight gain in HF diet-fed rats.

Studies have reported that dietary ginger improves lipid metabolism. Hot water extract of ginger improved the serum lipid profiles by lowering TG, TC, and LDL-C and increasing HDL-C in HF diet fed rats [13]. Additionally, supplementation of white ginger powder for 3 days reduced the plasma levels of TC, TG, VLDL-C, and LDL-C in cholesterol-enriched diet fed Wistar rats [14]. Likewise, HPG, in our study, improved the lipid profiles in serum and liver. Specifically, HPG enhanced fecal excretion of total lipids, TG and TC. These results indicate that HPG could exerts beneficial effects on lipid profiles in serum and liver in rats fed HF diet. In addition, it can be postulated that enhancement of lipid excretion by HPG could be one of the mechanisms that inhibit accumulation of lipids in serum and liver. 

To understand the mechanism underlying obesity-induced lipid metabolism and inflammatory response, we measured mRNA levels of the adipogenic genes and inflammatory cytokines in WAT. PPAR-γ is a ligand-activated transcription factor that acts on the differentiation and development of adipocytes [15]. aP2 is a marker protein for the mature adipocytes, involved in fat accumulation by acting on lipid biosynthesis pathways [15]. Chronic inflammation in obesity is manifested by abnormal expression of the genes that encode pro-inflammatory cytokines [16]. Weight loss, on the other hand, has been shown to alter the expression of genes involved in the production of cytokines in obesity [16]. TNF-α and IL-6 are pro-inflammatory cytokines synthesized when the lipid content increases in WAT, contributing to the pathogenesis of obesity-linked complications [1]. Adipocytes secrete chemotactic signals such as MCP-1, which trigger the recruitment of macrophages [1]. A study have reported that ethanol extract of ginger ameliorated obesity and inflammation through inhibition of adipose tissue accumulation and mRNA expression of pro-inflammatory cytokines such as IL-6 and TNF-α in WAT of mice fed high-fat diet [17]. In our study, mRNA levels of PPAR-γ and aP2 were reduced in the WEG and HPG groups. In addition, HPG reduced the mRNA levels of TNF-α, IL-6, and MCP-1. Thus, decreased adipogenic gene expression might explain the reduction of WAT mass by HPG, and HPG might have effects on the inhibition of inflammatory cytokine expression in WAT.

The pungent fragrance of the ginger is mainly attributed to volatile oils, primarily composed of gingerols and shogaols [18]. Among them, 6-gingerol is the major constituent of the gingerols along with 6-shogaol [18]. In our study, HPG showed excellent efficacy against weight loss and inflammation suppression compared with the conventional ginger extract. In a previous study, 6-gingerol showed anti-obesity effect by reducing lipid accumulation in mice fed a high-fat diet [19]. Further, rats fed a high-fat diet and treated with purified gingerol showed significant decrease in body weight and liver lipids compared to the control group [20]. An in vivo study has also reported that gingerol-and shogaol-enriched ginger extract increases fecal lipids including TG and TC [21]. Meanwhile, studies performed in vitro have reported that bioactive constituent of ginger such as 6-shogaol prevents adipogenesis and stimulates lipolysis in 3T3-L1 adipocytes [22]. In addition, 6-gingerol has been reported to block the action of PPAR-γ to prevent adipogenesis and the accumulation of cytoplasmic lipid droplets during the differentiation in 3T3-L1 preadipocytes as well as decreases the protein levels of fatty acid synthase and aP2 [23]. Moreover, 6-gingerol has been reported to inhibit TNF-α mediated c-Jun N-terminal kinases (JNK) phosphorylation, and 6-shogaol up-regulates PPAR-γ target gene expression in adipocytes [24]. Therefore, the amounts of 6-gingerol and 6-shogaol in both extracts were measured and compared to determine whether the superior effect of the HPG was due to increased 6-gingerol and 6-shogaol or not. As the data indicate, the amount of 6-gingerol in the HPG was significantly higher relative to that in the WEG, while total saponin and 6-shogaol amounts of the WEG and HPG were not statistically different, implying that 6-gingerol in HPG may have contributed in part to the inhibition of obesity and inflammation in rats fed the HF diet.

MicroRNAs (miRNAs) are highly conserved small non-coding RNAs that regulate gene expression at the post-transcriptional level. The miRNA binds to the complementary sequence of the target mRNAs to form a physical barrier or induce cleavage or degradation of the transcripts, thereby silencing the target transcripts. In particular, miRNAs are important regulators of development and function of adipose tissue. The expression of miR-21 increased during adipogenic differentiation in mesenchymal stem cells derived from the human adipose tissue [25]. Similarly, the expression of miR-21 increased in WAT, and correlated with the number of adipocytes in the epididymal fat [26]. Moreover, miR-132 has effects on activating NF-κB and transcription of IL-8 and MCP-1 in primary human pre-adipocytes and in differentiated adipocytes in vitro [27]. In this study, amelioration of adipogenic gene expression by ginger extract was accompanied by reduction of miR-21 expression in both WEG and HPG groups. The level of miR-132 was decreased in the HPG supplemented rats, and it was consistent with a decrease in the gene expression of cytokines such as TNF-α, IL-6, and MCP-1. Recently, Ahn et al. have reported that zerumbone, a cyclic sesquiterpene present in the rhizomes of wild ginger, ameliorates high-fat diet-induced adiposity by microRNA-146b/SIRT1-mediated adipogenesis [28]. However, the mechanism of regulation of ginger extracts on miRNA expression has not been reported so far. This study first demonstrated that ginger extracts regulate the expression of a variety of miRNAs such as miR-21 and miR-132 in WAT. Further, it is assumed that reduced miR-21 and miR-132 may be associated with post-translational regulation of genes related to adipogenesis and inflammation in rats fed a high-fat diet.

AMPK activation reduces lipogenesis and triglyceride synthesis, and prevents the expression and secretion of pro-inflammatory cytokines [29]. In a high-fat high-carbohydrate diet-fed rat model, ginger extract increased AMPKα phosphorylation and total AMPKα in skeletal muscle [30]. Hashem et al. have reported that 6-gingerol treatment in rats fed high-fat diet improves inflammatory state and metabolic disorders via targeting the AMPK-NF-κB pathway [31]. In the present study, AMPK activity was enhanced by the HPG supplementation in WAT of HF diet fed rats. Therefore, it appears that anti-obesity and anti-inflammatory effect of HPG is partially mediated by AMPK activation.

## 5. Conclusions

Our findings suggest that HPG inhibits body weight gain and adipose tissue in rats fed the HF diet. Additionally, HPG reduces lipid levels in serum and liver, promotes lipid excretion through feces, and causes down-regulation in the mRNA expression of genes related to adipogenesis and pro-inflammatory cytokines. Therefore, we concluded that HPG would be useful for application as a functional food for the prevention of obesity and inflammation. Specifically, this study has first reported that HPG inhibits miR-21/132 expression and activates AMPK in adipose tissue. Thus, further studies are needed to investigate the target-signaling pathways due to miR-21/132 down-regulation and AMPK activation by HPG. 

## Figures and Tables

**Table 1:** Effects of WEG and HPG on physiological variables.

Variables	HF	HF + WEG	HF + HPG
Initial body weight (g)	73.25 ± 1.33	73.66 ± 1.26	72. 46 ± 1.30
Final body weight (g)	472.64 ± 9.49 ^a^	438.31 ± 10.36 ^ab^	418.25 ± 11.98 ^b^
Body weight gain (g)	399.40 ± 9.32 ^a^	364.65 ± 9.46 ^ab^	345.79 ± 13.16 ^b^
Food intake (g/day)	18.15 ± 0.25	16.97 ± 0.37	17.06 ± 0.41
Food efficiency (g gained/g consumed)	0.301 ± 0.006 ^a^	0.293 ± 0.002 ^a^	0.278 ± 0.005 ^b^
Energy intake (kcal/day)	84.23 ± 1.16	78.75 ± 1.73	79.15 ± 1.91
Energy efficiency (g gained/kcal consumed)	0.065 ± 0.001 ^a^	0.063 ± 0.000 ^ab^	0.060 ± 0.001 ^b^

Values are expressed as mean ± SEM (*n* = 9/group). ^a,b^ Mean values with unlike superscript letters are statistically different at *p* < 0.05. HF: high fat; WEG: hot water extract of ginger; HPG: high-hydrostatic pressure extract of ginger.

## Supplementary Materials

The following are available online at http://www.mdpi.com/2072-6643/10/11/1567/s1. Table S1: compositions of experimental diets, Table S2: primers used for quantitative real-time PCR.
