# Supplementary material for: Ginger Extract Ameliorates Obesity and Inflammation via Regulating MicroRNA-21/132 Expression and AMPK Activation in White Adipose Tissue
Source: Nutrients. 2018 Oct 23;10(11):1567. doi: 10.3390/nu10111567 (PMC6266584; doi:10.3390/nu10111567)
Supplement: Supplementary file 1 [file nutrients-10-01567-s001.pdf]

**Supplementary Table S1**

Compositions of experimental diets (units: g/kg diet).

| Component | HF | HF + WEG | HF + HPG |
|---|---|---|---|
| Casein | 238.79 | 238.79 | 238.79 |
| Corn starch | 185.11 | 177.11 | 177.11 |
| Dextrose | 157.60 | 157.60 | 157.60 |
| Sucrose | 59.70 | 59.70 | 59.70 |
| Cellulose | 59.70 | 59.70 | 59.70 |
| Soybean oil | 29.85 | 29.85 | 29.85 |
| Lard | 208.94 | 208.94 | 208.94 |
| TBHQ | 0.02 | 0.02 | 0.02 |
| Mineral Mix [1] | 41.79 | 41.79 | 41.79 |
| Vitamin Mix [2] | 11.94 | 11.94 | 11.94 |
| L-Cystine | 3.58 | 3.58 | 3.58 |
| Choline bitartrate | 2.98 | 2.98 | 2.98 |
| WEG | - | 8.00 | - |
| HPG | - | - | 8.00 |
| Total | 1,000 | 1,000 | 1,000 |
| Fat, % (calories) | 45 | 45 | 45 |

[1] AIN-93G Mineral Mix. [2] AIN-93G Vitamin Mix. The experimental diets were formulated with slight modification based on the D12541 diet composition (Research Diets). HF, high-fat diet; HF + WEG, high-fat diet with 0.8% hot water extract of ginger; HF + HPG, high-fat diet with 0.8% high hydrostatic pressure extract of ginger; TBHQ, tert-butylhydroquinone.

**Supplementary Table S2**

Primers used for quantitative real-time PCR.

| Name | GenBank No. | Primer sequence (5’-3’) | Amplicon size (bp) |
|---|---|---|---|
| aP2 | NM_053365 | F: TCACCCCAGATGACAGGAAA<br>R: CATGACACATTCCACCACCA | 140 |
| β-actin | NM_031144 | F: GGCACCACACTTTCTACAAT<br>R: AGGTCTCAAACATGATCTGG | 123 |
| IL-6 | NM_012589 | F: ATAGTCCTTCCTACCCCAAC<br>R: TGCCGAGTAGACCTCATAGT | 143 |
| MCP-1 | NM_031530 | F: ACTCACCTGCTGCTACTCAT<br>R: CTACAGCTTCTTTGGGACAC | 101 |
| TNF-α | NM_012675 | F: CCCCTTTATCGTCTACTCCT<br>R: ACTACTTCAGCGTCTCGTGT | 139 |
| PPAR-γ | NM_001145366 | F: TGTGGGGATAAAGCATCAGG<br>R: CAAGGCACTTCTGAAACCGA | 175 |

aP2, adipocyte protein 2; IL-6, interleukin-6; MCP-1, monocyte chemoattractant protein-1; PPAR-γ, peroxisome proliferator-activated receptor-γ; TNF-α, tumor necrosis factor-α.
